# Evaluation of residual submicroscopic *Plasmodium falciparum* parasites 3 days after initiation of treatment with artemisinin-based combination therapy

**DOI:** 10.1186/s12936-020-03235-3

**Published:** 2020-04-21

**Authors:** Richard Mwaiswelo, Bill Ngasala

**Affiliations:** 1 Department of Microbiology, Immunology and Parasitology, Hubert Kairuki Memorial University, Dar es Salaam, Tanzania; 2 Department of Parasitology and Medical Entomology, Muhimbili University of Health and Allied Sciences, Dar es Salaam, Tanzania

**Keywords:** Submicroscopic, Parasites, Day 3, Artemisinin-based combination therapy, Transmission, Resistance

## Abstract

*Plasmodium falciparum* resistance against artemisinin has not emerged in Africa; however, there are reports of the presence of polymerase chain reaction-determined residual submicroscopic parasitaemia detected on day 3 after artemisinin-based combination therapy (ACT). These residual submicroscopic parasites are thought to represent tolerant/resistant parasites against artemisinin, the fast-acting component of the combination. This review focused on residual submicroscopic parasitaemia, what it represents, and its significance on the emergence and spread of artemisinin resistance in Africa. Presence of residual submicroscopic parasitemia on day 3 after treatment initiation leaves question on whether successful treatment is attained with ACT. Thus there is a need to determine the potential public health implication of the PCR-determined residual submicroscopic parasitaemia observed on day 3 after ACT. Robust techniques, such as in vitro cultivation, should be used to evaluate if the residual submicroscopic parasites detected on day 3 after ACT are viable asexual parasites, or gametocytes, or the DNA of the dead parasites waiting to be cleared from the circulation. Such techniques would also evaluate the transmissibility of these residual parasites.

## Background

Artemisinin-based combination therapy (ACT) is one of the major malaria control tools that have played a significant role in the reduction of malaria incidences globally [1–3], with some areas such as Zanzibar reaching the elimination stage [4]. *Plasmodium falciparum* resistance against artemisinins has however, emerged in parts of Southeast Asia (SEA) threatening the long-term use of ACT [5–7]. The resistance is associated with polymorphisms at propeller domain of the Kelch 13 protein encoded by the *P. falciparum pfk13* (or *k13* for short) gene of the parasite, and is expressed phenotypically as a prolonged microscopy-determined parasite clearance time [7, 8]. However, the *pfk13* gene mutations linked to resistance to artemisinins in SEA are not found in Africa, thus an in vivo microscopy-determined asexual parasites clearance time remains a reference method for the surveillance of resistance [9].

Resistance to anti-malarial drugs has often threatened malaria elimination efforts and historically has led to the resurgence of malaria incidences and deaths particularly in children < 5 years of age in sub-Saharan Africa (SSA) [10, 11]. Despite the recent case reports [12, 13], no artemisinin resistance has been confirmed in Africa [14–16]. Nevertheless, polymerase chain reaction (PCR)-determined residual submicroscopic parasitaemia has been reported on day 3 after case management using ACT in different parts of Africa [17–21]. Importantly, it is not well understood whether these residual submicroscopic parasites represent a viable resistant parasite population [19, 21, 22], and what role they play in determining treatment outcomes, transmission dynamics and spread of artemisinin resistance [17, 19].

The World Health Organization (WHO) has approved five artemisinin-based combinations, including artemether–lumefantrine, artesunate-amodiaquine, artesunate-sulfadoxine–pyrimethamine, artesunate–mefloquine and dihydroartemisinin-piperaquine [23]. Artemether-lumefantrine is the most widely used ACT medicine in Africa [24]. This review focused on the current knowledge on the prevalence and significance of day 3-PCR determined residual submicroscopic parasites following treatment with ACT on malaria transmission dynamics and spread of artemisinin resistance. Published reports on the treatment of uncomplicated *P. falciparum* malaria using artemether-lumefantrine, artesunate-amodiaquine, artesunate–sulfadoxine-pyrimethamine, dihydroartemisinin-piperaquine and a new drug artesunate-pyronaridine [25], were included in the review.

## Parasite clearance time following ACT

The major characteristic of ACT is its ability to rapidly clear asexual *P. falciparum* parasites [26–29]. ACT consists of fast acting artemisinin derivatives and slow-acting non-artemisinin partner drugs [26, 28, 29] (Table 1). The artemisinin component rapidly clears a large biomass of malaria parasites achieving parasite reduction on the order of 10^8^-fold reduction within 3 days, before the long-acting partner drug has cleared the residual parasitaemia [26, 30, 31]. Artemisinins act on asexual parasites from early young ring stages to mature trophozoites, rapidly clearing the parasites [30]. The accelerated clearance of young ring-stage parasites prevents further maturation and sequestration of trophozoites, which is associated with the severity of the infection [29, 31]. The rapid parasite clearance rate therefore, not only optimize the therapeutic benefits, but also protects both components of the combination and minimizes the risk of emergence and spread of resistant parasite populations [10]. ACT also act on young gametocytes, reducing gametocytes carriage and thus diminishes the transmission potential of the treated infection [29, 32]. However, parasite clearance following treatment with anti-malarial drug is influenced by various host, parasite and drug factors [33, 34].

**Table 1:** Artemisinin-based combination therapies used in Africa

Artemisinin-based combination therapy	Artemisinin component	Partner drug	Partner drug target	Presence of persistent submicroscopic parasites on day 3 after treatment	Validated marker of drug resistance
Artemether-lumefantrine	Artemether	Lumefantrine	Interferes with haem detoxification	Yes [17, 19–21, 25, 86]	*pf*mdr1 N86, 184F, D1246 Amplification of *pf*mdr1 copy number *pf*crt K76 Haplotype CVMNK [24, 48, 87, 88]
Artesunate-amodiaquine	Artesunate	Amodiaquine	Interferes with haem detoxification	Yes [89]	*pf*mdr1 86Y, Y184, 1246Y *pf*crt 76T Haplotype SVMNT [24, 87]
Artesunate–sulfadoxine-pyrimethamine	Artesunate	Sulfadoxine Pyrimethamine	Inhibits two enzymes involved in folate biosynthesis pathways. Sulfadoxine inhibits dihydropteroate synthase (dhps), and Pyrimethamine inhibits dihydrofolate reductase (dhfr) [90, 91]	Yes [90]	*pf*dhfr 51I, 59R, 108 N *pf*dhps 437G, 540E [24, 90, 91]
Dihydroartemisinin-piperaquine	Dihydroartemisinin	Piperaquine	Not well understood but linked to inhibition of haem degradation pathways	Yes [17]	Plasmepsin II and III amplification [24, 88]
Artesunate-pyronaridine (Registered and in use in some countries [23])	Artesunate	Pyronaridine	Interferes with haem detoxification	Yes [25]	*pf*crt 76T [24]

## Prolonged parasite clearance time as a measure of artemisinin resistance

Parasite clearance time is a robust measure of efficacy of anti-malarial drugs [35]. Prolonged parasite clearance time following treatment with chloroquine, sulfadoxine-pyrimethamine and mefloquine was shown previously to be a first indicator of resistance against the drugs [36, 37]. Prolonged clearance time following ACT is, therefore, viewed as first evidence of reduced susceptibility of the parasites to artemisinin and possibly preceding the clinically significant resistance [5, 31, 33, 35]. The proportion of patients with parasitaemia on day 3 following ACT is a routine indicator in monitoring *P. falciparum* sensitivity to artemisinin and its derivatives [23, 38], rather than the long-acting partner drug [29]. Partial artemisinin resistance can either be suspected or confirmed. Suspected resistance to artemisinin is described as an increase in parasite clearance half-life to ≥ 5 h, as evidenced by ≥ 10% cases with microscopy-determined parasites on day 3 after initiation of treatment with an artemisinin-based combination [23, 39], or high prevalence of *pfk13* mutants [9, 23, 39]. The confirmed artemisinin resistance occurs when there is a combination of delayed parasite clearance and *pfk13* resistance-validated mutations for the same patient [38]. Importantly, there is no evidence of full artemisinin resistance; nevertheless, partial artemisinin resistance may facilitate the selection for the partner drug resistance. On the other hand, the classical drug resistance is when a parasite that normally would have been cured by a treatment regimen survives, multiplies and is transmitted to a new host [33]. Interestingly, in Africa microscopy-determined parasite clearance time is rapid following ACT, with very few patients having parasitaemia on day 3 [19, 35, 40–42]. However, clearance slopes in infections in SSA are likely to underestimate inherent resistance of parasites due to greater host immunity in high-transmission settings [43]. Presence of residual submicroscopic parasitaemia on day 3, therefore, provides the early warning signs of the emergence of *P. falciparum* resistance against artemisinin-based combinations in Africa (Table 1).

## Residual submicroscopic parasitaemia on day 3 after ACT

The PCR-determined submicroscopic parasites are those that are below the detection limit of a standard light microscopy, but can be detected by a much more sensitive tool, the PCR [44]. Residual PCR-determined submicroscopic malaria parasites include both asexual and sexual parasites remaining days after initiation of anti-malarial treatment. ACT has continued to achieve rapid microscopy-determined parasitological clearance across SSA, with a majority of treated patients being cleared of asexual parasites within 48 h of treatment initiation [3, 35, 45–47]. More precisely, over 90% of patients are being microscopically parasite negative by day 2 and 99% by day 3 post-initiation of ACT [35, 46, 47]. The PCR on the other hand, has revealed the presence in SSA of *P. falciparum* genotypes that are probably more likely to survive ACT at submicroscopic level (Table 1), and probably contribute to an onward transmission and subsequent patient recrudescence [17, 19–21, 48]. Studies in Angola [20], Kenya [17], Tanzania [18, 21], and Uganda [19] have reported substantial proportions of individuals treated with artemisinin-based combination, with residual submicroscopic parasitaemia between days 3 and 7. Whereas a study in Angola has reported an increase, over time, in the proportion of patients with residual submicroscopic parasitaemia on day 3 [20], a study in Tanzania reported an increase followed by a decline in the proportion of patients with residual parasitaemia across years of surveillance [21]. Several submicroscopic parasite subpopulations have also been observed in Tanzania with no known *pfk*-*13* resistance-associated mutations, however, they clear as slowly as parasites in Cambodia, which are labeled drug resistant and do harbour the mutations [43]. Interestingly, these parasites in Tanzania with similar prolonged clearance as those in Cambodia were from a study conducted in 2006 at a time when artemether-lumefantrine was not yet adopted as first-line treatment in Tanzania, and thus were probably naive of the drug [21, 43]. Nevertheless, after nearly two decades of a wide-scale use of ACT in Africa, the combination therapy has remained efficacious despite the presence of residual submicroscopic parasitaemia on day 3 [3, 16, 17, 19–21]. The questions remaining to be answered are, therefore, what are the factors other than parasite tolerance/resistance that may determine the presence of residual submicroscopic parasitaemia after ACT and whether residual PCR-determined submicroscopic parasitaemia represents viable asexual parasites.

## Factors associated with the presence of residual submicroscopic parasitaemia

Apart from drug susceptibility, several factors affect the clearance of microscopy-determined parasitaemia following treatment with anti-malarial drugs leading to a large variation in clearance rates of sensitive and resistant parasite strains [9]. Parasite clearance as a predictive value for artemisinin resistance is influenced by factors including pre-treatment parasitaemia, parasite strains, parasite developmental stage, host immunity, partner drug efficacy, sample size, quality of microscopy, and the pharmacokinetic/pharmacodynamics profile of the different artemisinin derivatives and the partner drugs [9, 34, 35]. For instance, following anti-malarial treatment individuals with low pre-treatment parasitaemia clear parasitaemia more rapidly than those with higher parasitaemia. On the other hand, the burst of mature schizonts releases thousands of merozoites into the circulation increasing parasitaemia level in the peripheral blood [34]. Furthermore, sequestration of trophozoites to the inner vessels starting at around 18 h of the 48-h asexual life cycle leads to a sudden decline in the level of parasitaemia in the peripheral blood.

Likewise, the same factors that affect the clearance rate of microscopy-determined parasitaemia have also shown to influence the presence of day 3 residual submicroscopic parasitaemia. Studies in Kenya and Tanzania have shown the association of the presence of residual submicroscopic parasitaemia on day 3 after initiation of ACT with higher pre-treatment microscopy-determined asexual parasitaemia, anaemia, age < 5 years, and fever at baseline [17, 21]. Of the factors, host immunity plays a critical role in the clearance of parasites, including resistant *P. falciparum* infections. In areas of intense transmission, acquired immunity develops at a relatively young age, and is a key determinant of the anti-malarial therapeutic response [35]. Partially immune individuals may, therefore, experience some response to drug treatment even if they are infected by drug-resistant parasites. For instance, a study in Mali has shown older children to be able to more effectively clear resistant parasites than younger children [49]. Similarly, following anti-malarial treatment, immunity is the primary determinant of clearance rate [50]. However, other studies have found no association between baseline characteristics and the presence of residual submicroscopic parasitaemia on day 3 [19].

The pharmacodynamics and pharmacokinetics of the drugs may also play a significant role in the clearance rate of malaria parasites. The fast acting component artemisinin, has a half-life of 1-2 h, clearing rapidly from the body [26, 28]. Thus, a 3-day ACT regimen provides anti-malarial activity for two asexual parasite cycles and results in a reduction of parasitaemia in the infected individual by a factor of about 100 million parasites, but this still leaves up to 100,000 parasites for the long-acting partner drug to remove, variably assisted by the immune response [29]. Of note, the day 3 residual submicroscopic parasites are assessed at the time when the fast-acting artemisinin derivatives have already been cleared from the body; therefore, the long-acting partner drug is virtually acting alone. The day 3 PCR-determined residual parasites might therefore, be representing a remaining fraction of parasitaemia to be cleared by the long-acting partner drug after the short-acting artemisinin component has already been cleared. The observation is substantiated by the presence of residual parasitaemia in a study conducted in 2006 in Bagamoyo, Tanzania, using artemether-lumefantrine, at a time when the combination therapy was not a first-line treatment policy in the country [18, 21, 43]. At that time most of the parasites were carrying *P. falciparum* multidrug resistance-1 (*mdr1)* 86-Tyrosine (Y) and *P. falciparum* chloroquine resistance transporter (*crt)* 76-Threonine (T) alleles, but were known to be sensitive to artemether-lumefantrine [21]. Malaria prevalence at the time was also high in the study area, therefore, the partial immunity against malaria could be acquired at a very young age, and the study was conducted in children aged 6 months to 10 years [18]. The nearly equal proportions of submicroscopic parasitaemia on day 3 between children aged < 5 years and the older children in the Bagamoyo study [21], probably indicates the influence of other factors for the presence of residual parasitaemia on day 3, including selective immunity whereby some parasite populations are not affected by both the acquired immunity and drug effect [43]. Nonetheless, even though the above-mentioned factors are found to influence the presence of submicroscopic parasitaemia on day 3, more studies are needed to evaluate the viability of the detected residual asexual parasites.

## Efficacy of ACT in relation to day 3 residual submicroscopic parasitaemia

Failure to rapidly clear the asexual parasite biomass by the fast-acting artemisinin component of the combination is thought to cause more parasites to be exposed to the long-acting partner drug alone increasing the risk of selection to the partner drug [38]. Prolonged parasite clearance increases the chances of more resistant asexual parasites to differentiate into gametocytes and then be transmitted after mosquito bite. Despite the presence of substantial proportion of submicroscopic parasitaemia on day 3 after ACT, in Africa the various combination therapies have remained highly efficacious [3, 16, 51], with the PCR-corrected efficacy reported to be above 90% in the surveyed areas [17, 19–21, 47, 52]. Similar better treatment outcomes are reported in Myanmar and Vietnam despite the presence of prolonged clearance half-life of more than 5 h, ≥ 10% of ACT-treated patients with microscopic-determined asexual parasites, selection of *pfk13* [53, 54], and residual submicroscopic parasitaemia twice as much as those with microscopy-determined parasitaemia [53]. It is, therefore, clear that delayed parasite clearance does not necessarily lead to treatment failure [38]. However, in Kenya the PCR-detected submicroscopic parasitaemia on day 3 was statistically significantly associated with PCR-adjusted recrudescence [17], but the significant association occurred after the standard WHO guideline for differentiation of recrudescence from new infection was modified [55]. Importantly, treatment failure rates following ACT outside the Great Mekong Sub-region occurs in the absence of artemisinin resistance, and are mainly due to partner drug resistance [56], although a cross-resistance cannot be ruled out. Treatment failure rate equal to or above 10% should prompt a change in the national anti-malarial treatment policy [38].

## Selection of markers of drug resistance in residual submicroscopic parasites

Molecular markers of drug resistance are among the tools widely used for surveillance of drug sensitivity [57, 58]. Specific polymorphisms within the *P. falciparum* multidrug resistant (*pfmdr1*) and *P. falciparum* chloroquine resistance transporter (*pfcrt*) genes are widely used as markers for surveillance of ACT resistance in Africa [59–65], where *pfk13* mutations are present but their variants differ from those in SEA and their correlation with artemisinin resistance has yet to be substantiated [14, 15, 35, 66]. Both *pfmdr1* and *pfcrt* resistant haplotypes are saturated in the parasite population following years of wide-scale use of ACT, and are being detected both at baseline and in residual submicroscopic parasites detected on day 3 following ACT [21] (Table 1). Five *pfmdr1* amino acid positions asparagine (N)-86, tyrosine (Y)-184, serine (S)-1034, N1042, and aspartic acid (D)-1246 influence susceptibility to lumefantrine, quinine, artemisinins and mefloquine [11]. Selection of *pfmdr1* haplotypes NFD (N86Y, F184Y and D1246Y) has been consistently observed after artemether-lumefantrine treatment in Africa, and is associated with decreased parasite susceptibility to the arylaminoalcohol quinolone long-acting partner drugs, including lumefantrine [59]. On the other hand, selection of haplotype YYY is linked to decreased sensitivity to 4-aminoquinoline drugs, such as chloroquine and amodiaquine [59]. Amplification of *pfmdr1* copy numbers is associated with reduced susceptibility to artemisinins, lumefantrine, and mefloquine [11]. Likewise, selection of *pfcrt* haplotypes influence susceptibility to the commonly used partner drugs in Africa, lumefantrine and amodiaquine [11, 61]. The *pfcrt* 76T is associated with amodiaquine tolerance [11], whereas lysine (K)-76 reduces susceptibility to lumefantrine [21, 60, 61, 67].

While some studies have indicated evidence of statistically significant within-host directional selection of *pfmdr1* NFD and *pfcrt* CVMNK haplotypes on day 3 post-ACT [19, 48, 68], other studies have indicated the absence of statistically significant directional selection between at baseline and on day 3 [21] or at baseline and among recurrent infections [69] over time of both *pfmdr1* and *pfcrt* haplotypes. Random fluctuation for instance, of *pfcrt* is observed between day 0 and day 3, from T76 to K76 or mixed infection K76T; or from K76 to mixed infection K76T or 76T; or from mixed infection K76T to K76 or 76T and vice versa [21]. Similarly, there has been fluctuation of *pfmdr1* from 86Y to N86 or mixed infection N86Y; or from N86 to 86Y or mixed infection N86Y; or from mixed infection N86Y to N86 or 86Y and vice versa [21]. The lack of directional selection in some study areas is partly probably due to saturation of the parasite population, particularly with *pfmdr1* N86 and *pf*crt K76 in these areas [21, 60]. Importantly however, the *pfcrt* CVMNK and *pfmdr1* NFD haplotypes found in African residual submicroscopic parasites have not been directly linked to microscopy-determined delayed parasite clearance time in SEA parasites [70]. On the other hand, carriage of CVMNK prior to treatment is associated with risk of parasite recurrence on days 28 or 42 for AL, whereas the presence of NFD haplotype is not associated with risk of recrudescence [48]. However, a study in Tanzania has indicated the absence of association between both baseline and on day 3 selection of *pfmdr1* and *pfcrt* with recurrent infection [21]. It is worth noting that the markers of drug resistance are established when drug resistance has already developed [58].

No artemisinin resistance has been detected in Africa, and there is only partial artemisinin resistance in SEA. This might explain the lack of association between markers of drug resistance and treatment outcomes. Adequate clinical and parasitological response is observed in SEA after ACT despite the presence of parasites with *pfk13* mutations [53, 54]. Therefore, while molecular markers of drug resistance are very important for the surveillance of anti-malarial resistance, it should be understood that they are not often predictive of clinical resistance [31]. However, the potential to detect emerging trends of drug resistance before outright clinical failure makes molecular markers useful tools [31].

## Viability of residual submicroscopic asexual parasites

The asexual parasite is the only stage of the malaria parasite responsible for pathogenesis and treatment failure. The asexual parasites can also under certain circumstances differentiate into a transmissible gametocyte stage. After ACT, a fraction of patients may continue to harbour viable microscopy-determined parasites that persist as chronic infections [19]. These tolerant or resistant asexual parasites that survives the anti-malarial drug pressure may also differentiate into gametocytes and be transmitted to the mosquito vector, where they replicate and expand the tolerant/resistant parasite population. However, the recent observation of residual submicroscopic parasitaemia 3 days after initiation of a full course treatment with an artemisinin-based drug combination [19, 71] leads to question whether these residual parasites represent viable resistant asexual parasites, which could contribute to the spread of anti-malarial drug resistance.

PCR-based detection techniques are more sensitive than microscopy [72], being able to detect as little as 2 parasites per microlitre of blood [44]. PCR uses specific primers to detect *P. falciparum* DNA targets and RNA transcripts of 18S rRNA or cytochrome b [73–76], abundantly present in both asexual parasites and gametocytes [71]. Gametocytes commonly persist at low concentrations for several weeks after ACT or after ACT plus primaquine [17, 71]. Post-treatment persistence of 18S DNA or RNA in peripheral blood can thus be a consequence of the presence of both trophozoites and gametocytes. Thriemer et al., have shown that patients who are qPCR positive for malaria parasites at day 7 are more likely to have been carrying gametocytes before treatment and during follow-up [53]. Ring-stage RNA has also been employed to assess residual submicroscopic parasitaemia, however, the transcripts are not exclusively expressed in the ring-stage, and it is not clear whether they represent viable asexual ring-stage parasites [19]. Other ring-stage markers SBP1, REX1 and PHISTb have been used to assess submicroscopic parasitaemia on day 3 [71]. The SBP1 is most sensitive for asexual ring-stage parasites, but is also most intensely expressed in stage V gametocytes [71]. On the other hand, REX1 and PHISTb markers show high specificity for ring-stage trophozoites, but their specificity is compromised at gametocyte densities > 10^6^ gametocytes/μL, and also have lower sensitivity than SPB1 [71]. It is also not clear whether the ring-stage trophozoites detected on day 3 by these markers represent viable malaria parasites or whether mRNA transcripts may linger in the circulation after asexual parasites have been cleared [71].

Malaria parasite nuclear materials are removed from the circulation by the circulating and reticuloendothelial phagocytes [50, 77, 78]. ACT also activate the spleen to remove damaged and dead parasites from circulating infected erythrocytes through pitting, therefore, facilitating the rapid clearance of asexual parasites [30, 77–79]. Thus, DNA derived from dead parasites circulates for less than 48 h [71, 73]; and it is thought that non-viable cells are unlikely to give PCR positive signals, therefore, PCR analysis of malaria-infected blood accurately reflects the presence of live parasites [73]. Conversely, live but drug-damaged parasites that would be unable to initiate an infection when re-inoculated may also contribute to positive PCR signals [50, 71]. It is, therefore, still unclear whether the observed residual PCR-determined submicroscopic parasitaemia represents viable resistant asexual parasite strains, or are drug-damaged parasites waiting to be cleared, or are parasites remaining after the clearance of the rapid acting artemisinins and will be cleared by the long-acting partner drug.

## Transmission potential of residual submicroscopic parasitaemia

Mature stage V gametocytes, a form of malaria parasite that develops from asexual parasites, are the only stage responsible for transmission of the infection from human to a mosquito vector [80]. Immature stage (I-IV) gametocytes are sequestering away from the peripheral circulation in a manner similar to mature asexual stage parasites [81]. Artemisinins acts against young gametocytes, significantly reducing gametocytes carriage [31, 32, 82–84], but are not potent against mature stage V *P. falciparum* gametocytes. The rapid asexual parasite clearance rate exerted by artemisinins also reduces the number and window of asexual parasites to differentiate into gametocytes, therefore, reducing transmission [29, 30, 32, 82]. On the other hand, anti-malarial drug resistance is associated with increased treatment failure and gametocytes carriage. Residual submicroscopic parasitaemia are also thought to be associated with longer duration of gametocytes carriage, higher likelihood of infecting mosquitoes, a higher parasite burden in the mosquito [17], and thus further risk of infecting human hosts [48]. Mosquito membrane feeding experiments have also shown that patients with residual parasitaemia on day 3 are more likely to infect mosquito on the day of experiment (day 7) than those who have cleared asexual parasites by day 3 [17]. However, it is not known whether the PCR-determined parasites on day 3 represent viable asexual parasites that would later differentiated into gametocytes and are mature enough to be transmitted to mosquito on day 7, the day of transmission experiment. The development and maturation of gametocytes from stage I to V requires 10-12 days [81, 85], therefore, it is almost impossible for the asexual parasites detected on day 3 to differentiate into gametocytes and be ready for transmission within 4 days. Likewise, it is not clear whether viable drug-damaged asexual parasites can differentiate into gametocytes. This does also not rule out that the positive transmissions occurring on the day of experiment are due to submicroscopic gametocytes which were present in surveyed individuals prior to the initiation of ACT. It should be emphasized that the detected PCR signals based on asexual parasites’ 18 s rRNA are also present in gametocytes [71].

## Conclusion

Presence of residual submicroscopic parasitemia on day 3 after treatment initiation leaves question on whether successful treatment is attained with ACT. Thus there is a need to determine the potential public health implication of the PCR-determined residual submicroscopic parasitaemia observed on day 3 after ACT. Robust techniques, such as in vitro cultivation, should be used to evaluate if the residual submicroscopic parasites detected on day 3 after ACT are viable asexual parasites, or gametocytes, or the DNA of the dead parasites waiting to be cleared from the circulation. Such techniques would also evaluate the transmissibility of these residual parasites.

## Data Availability

Not applicable.

## Abbreviations

ACT: Artemisinin-based combination therapy
DNA: Deoxyribonucleic acid
MeSH: Medical subject headings
mRNA: Messenger ribonucleic acid
*pfcrt*: *Plasmodium falciparum* chloroquine resistance transporter
*pfmdr*: *Plasmodium falciparum* multidrug resistance
PCR: Polymerase chain reaction
qPCR: Quantitative polymerase chain reaction
RNA: Ribonucleic acid
SEA: Southeast Asia
SSA: sub-Saharan Africa
WHO: World Health Organization
